# Integrated Physical–Mechanical Characterization of Fruits for Enhancing Post-Harvest Quality and Handling Efficiency

**DOI:** 10.3390/foods14142521

**Published:** 2025-07-18

**Authors:** Mohamed Ghonimy, Raed Alayouni, Garsa Alshehry, Hassan Barakat, Mohamed M. Ibrahim

**Affiliations:** 1 Department of Agricultural and Biosystems Engineering, College of Agriculture and Food, Qassim University, Buraydah 51452, Saudi Arabia; m.elsayed@qu.edu.sa; 2 Department of Food Science and Human Nutrition, College of Agriculture and Food, Qassim University, Buraydah 51452, Saudi Arabia; r.alayouni@qu.edu.sa; 3 Department of Food Science and Nutrition, College of Sciences, Taif University, P.O. Box 11099, Taif 21944, Saudi Arabia; garsa.a@tu.edu.sa; 4 Department of Agricultural Engineering, Faculty of Agriculture, Cairo University, Giza 12613, Egypt; mohamed.ibrahim@agr.cu.edu.eg

**Keywords:** fruit, quality, compression, rupture, post-harvest quality, food supply

## Abstract

Quality and mechanical resilience are crucial for reducing losses in fruit production and for supporting food chains. Indeed, integrating empirical data with rheological models bridges gaps in fruit processing equipment design. Therefore, the objective of this research is to analyze the relationship between the mechanical and physical properties of seven economically important fruits—nectarine, kiwi, cherry, apple, peach, pear, and apricot—to assess their mechanical behavior and post-harvest quality. Standardized compression, creep, and puncture tests were conducted to establish mechanical parameters, such as rupture force, elasticity, and deformation energy. Physical characteristics including size, weight, density, and moisture content were also measured. The results indicated significant differences among the various categories of fruits; apples and pears were most suitable for mechanical harvesting and long storage periods, whereas cherries and apricots were least resistant and susceptible to injury. Correlations were high among the physical measurements, tissue firmness, and viscoelastic properties, thereby confirming structural properties’ contribution in influencing fruit quality and handling efficiency. The originality of this research is in its holistic examination of physical and mechanical properties under standardized testing conditions, thus offering an integrated framework for enhancing post-harvest operations. These findings offer practical insights for optimizing harvesting, packaging, transportation, and quality monitoring strategies based on fruit-specific mechanical profiles.

## 1. Introduction

Quality assessment of fruits is necessary for determining their appropriateness for market consumption, processing, and sale. In post-harvest assessment parameters, mechanical properties—especially those obtained from force–deformation curves—provide valuable information on the fruit’s texture, structural strength, and mechanical damage resistance during handling and transport [1,2]. Knowledge of fruits’ responses to mechanical stress is crucial for quality maintenance along the value chain and technological developments in sorting, packaging, and processing. Fruits’ integrated physical and mechanical characteristics are closely linked to their post-harvest quality and handling efficiency. Recent studies have demonstrated that attributes such as size, mass, moisture content, density, firmness, tensile strength, and modulus of elasticity change significantly throughout fruit development and storage, directly influencing susceptibility to mechanical damage and overall quality retention [3,4].

Mechanical damage, including bruising, cracking, and tissue breakdown, primarily occurs during harvesting, transportation, and storage and is strongly affected by the fruit’s physical and mechanical properties. For example, research has shown that as fruits ripen, they typically become softer and more prone to compression and impact injuries, accelerating quality loss and shortening shelf life [3,5]. Specific findings indicate that the degree of compression damage is determined by factors such as loading displacement, fruit morphology, and storage duration, with increased damage leading to greater mass loss and reduced firmness [6].

Quantitative parameters of mechanical force–deformation analysis are firmness, elasticity, rupture force, and deformation energy, essential indicators of fruit durability and storability. These parameters indicate internal anatomical and physical properties, including tissue density, cell wall integrity, and water content [7,8]. Also, surface characteristics like cuticle arrangement and peel integrity play a significant role in determining the mechanical properties of fruits since they impact stress distribution and failure locations [9]. The precise identification of these properties allows scientists and industry players to establish suitable mechanical resistance values for handling and automation processes. Fruits can be categorized based on mechanical response as brittle, elastic, or viscoelastic. These classes influence post-harvest behavior and consumer attitudes [10]. For instance, apples and pears typically exhibit elastic rupture behavior, while fruits like nectarines and apricots undergo more prolonged viscoelastic deformation before failure. Such differences underscore the need for fruit-specific mechanical evaluation. The demand for mechanical profiling is growing due to its utility in automation and quality control. Modern sorting systems increasingly depend on mechanical feedback to assess firmness and ripeness, reducing damage during processing [11]. Studies on apples, pears, peaches, nectarines, apricots, kiwis, and cherries have revealed distinct mechanical characteristics, emphasizing the need to tailor post-harvest strategies accordingly [12,13].

Moreover, the effectiveness of post-harvest handling systems—such as mechanized harvesting, packaging, and transport—relies on understanding these integrated characteristics. Studies employing non-destructive testing and predictive modeling have enabled more accurate damage detection and optimization of handling protocols, thereby reducing losses and maintaining fruit quality [6,9]. Recent studies confirm the relevance of mechanical data in predicting shelf life and mechanical injury. For example, Ghonimy et al. [3] showed that higher date rupture forces were associated with longer visual and textural preservation during storage. Likewise, Hassan et al. [14] highlighted the potential of mechanical metrics in guiding decisions related to fruit toughness. In addition, force–deformation analysis supports quality assurance during slicing, drying, juicing, and freezing, where mechanical behavior directly impacts process performance and product quality [15]. The selection of the fruit types—nectarine, kiwi, cherry, apple, peach, pear, and apricot—was based on their broad commercial relevance and structural diversity. These fruits represent different botanical classes, such as pome fruits (apple, pear), stone fruits (peach, nectarine, apricot, cherry), and soft-fleshed berries (kiwi), each exhibiting distinct anatomical features including pit presence, peel thickness, and tissue composition. Their inclusion ensures representation of a wide range of mechanical responses, from firm and elastic to soft and viscoelastic textures. Furthermore, all selected fruits are commonly grown in Mediterranean and semi-arid climates and are subject to similar post-harvest challenges. This makes them suitable models for developing generalized handling and quality control strategies applicable to a broad spectrum of fruit types. It is important to note that while certain fruits such as kiwi and apple differ significantly in their anatomical structure and tissue classification, their inclusion was intentional to allow meaningful comparisons across a diverse mechanical spectrum. The objective was not to equate their internal morphology, but to evaluate how structurally distinctly fruits behave under identical mechanical testing protocols. The mechanical behavior of these fruit types has been previously investigated in various studies, supporting their relevance to post-harvest handling research. For instance, nectarines and peaches have been characterized as susceptible to bruising and rapid softening, with low rupture thresholds and high viscoelastic deformation under compression [16,17]. Apples and pears, in contrast, exhibit high firmness and elastic behavior, making them suitable for automated handling and storage [6,18]. Kiwifruit is often studied for its unique combination of stiff outer skin and soft interior, with mechanical response strongly influenced by maturity and turgor pressure [19]. Cherries and apricots, which are highly sensitive to external stress, demonstrate low rupture energy and require gentle manual handling, as noted in recent evaluations of firmness and skin integrity [20,21].

The main finding is that optimizing post-harvest quality and handling efficiency requires a comprehensive assessment of fruits’ integrated physical–mechanical properties, as these directly determine their resilience to mechanical stresses and their capacity to retain quality during the post-harvest period [3,4,6]. Thus, this study aimed to evaluate the physical and mechanical properties of seven commercially important fruit types—nectarine, kiwi, cherry, apple, peach, pear, and apricot—under controlled loading conditions, in order to highlight the relevance of these properties to post-harvest quality and to provide a basis for improving handling, storage, and processing practices.

## 2. Materials and Methods

### 2.1. Fruit Samples and Experimental Design

Fruit samples from seven different types—nectarine (*Prunus persica* var. nucipersica cv. Big Top), peach (*Prunus persica* cv. Florida Prince), kiwi (*Actinidia deliciosa* cv. Hayward), cherry (*Prunus avium* cv. Bing), apple (*Malus domestica* cv. Anna), apricot (*Prunus armeniaca* cv. Canino), and pear (*Pyrus communis* cv. Le Conte)—were collected at commercial maturity from orchards located in key fruit-producing regions of Egypt. Special attention was given to standardizing harvest timing, selecting market-quality fruits, and ensuring consistent post-harvest handling across all fruit types. Hand-picked nectarine samples of the ‘Big Top’ variety were collected from commercial orchards in the El-Nubaria area of the Beheira Governorate (30.6219° N, 30.1792° E), Egypt, with sandy loam soil types and a semi-arid climate. The orchards are irrigated using drip irrigation systems and have high solar radiation, with summer temperatures rising to 40 °C and an annual rainfall amount that typically never exceeds 100 mm. Fruits were harvested in the early morning to minimize field heat and placed in ventilated plastic crates positioned under the shade to minimize degradation before analysis. Peach fruits (cv. Florida Prince) were collected from the same orchards in El-Nubaria, which are optimized for early-season peach production. Well-drained soils, low humidity, and efficient irrigation support high-quality fruit development. Hand-picking was performed early in the morning, with immediate shading and crating for transport. Kiwi samples (cv. Hayward) were obtained from an experimental orchard in El-Nubaria (30.6219° N, 30.1792° E), Egypt, where supplemental irrigation and organic soil amendments support limited-scale kiwi cultivation. Although kiwi typically requires heavy chilling hours, certain microclimatic situations in El-Nubaria, such as cold winter nights, enable successful production. Fruits were hand-picked, padded with soft packaging to prevent bruising, and transported to the Agricultural Engineering Department, Faculty of Agriculture, Cairo University, Egypt, within 6 h of harvesting. Sweet cherry fruits (cv. Bing) were collected from a pilot orchard in El-Nubaria (30.6219° N, 30.1792° E), Egypt, where trial cultivation of sweet cherry is conducted under shaded net structures and precision irrigation. While Prunus avium typically thrives in cooler climates, this pilot farm leverages localized cooling strategies and selective cultivars. Fruits were harvested manually using gloves to prevent damage, precooled on-site to 5–7 °C, and transported in insulated containers to the laboratory within 24 h. Apple samples (cv. Anna) were sourced from commercial orchards in El-Nubaria. The Anna cultivar, known for its adaptation to warm climates, is well-suited to the region’s loamy sand soil and high summer temperatures. Orchards use drip irrigation and integrated pest management practices.

Fruits were picked at maturity, packed in ventilated containers, and maintained at approximately 10 °C during transportation to preserve firmness. Apricot fruits (cv. Canino) were harvested from the same area, benefiting from the deep, well-drained soils of El-Nubaria. Canino apricots are valued for their firmness and suitability for fresh market distribution. Harvesting was performed manually to minimize mechanical damage, and the fruits were cushioned in perforated food-grade containers for transit. Pear samples (cv. Le Conte) were collected from commercial orchards in Ismailia Governorate (30.5830° N, 32.2650° E), Egypt. The Le Conte cultivar is commonly grown in Egypt due to its tolerance to warm climates and resistance to local pests. Orchards in this region utilize sandy soils, drip irrigation, and integrated pest management. Fruits were picked at commercial maturity, packed in ventilated containers, and transported under cooled conditions (around 10 °C) to maintain post-harvest quality. All fruit samples were carefully selected to be healthy and free from visible defects, diseases, or pest damage. For each fruit type, 60 uniform fruits were selected for analysis. Harvest timing was coordinated across all locations to align with the commercial ripening stage, determined based on color development, firmness, and soluble solids content in accordance with national agricultural standards.

Fruits were delivered to the Biological and Environmental Engineering System Laboratory, Agricultural Engineering Department at the Faculty of Agriculture, Cairo University, Egypt, within 6 to 24 h after harvest, depending on the orchard location. During transportation, fruits were stored in ventilated crates placed inside insulated boxes, with internal temperatures maintained between 12 °C and 14 °C and relative humidity between 85% and 90%, verified using data loggers. Upon arrival, samples were sorted, labeled, and conditioned for 24 h at 23 ± 1 °C and 50 ± 5% relative humidity to allow moisture equilibrium prior to mechanical testing.

### 2.2. Physical Properties of Fruits

**Figure 1:**
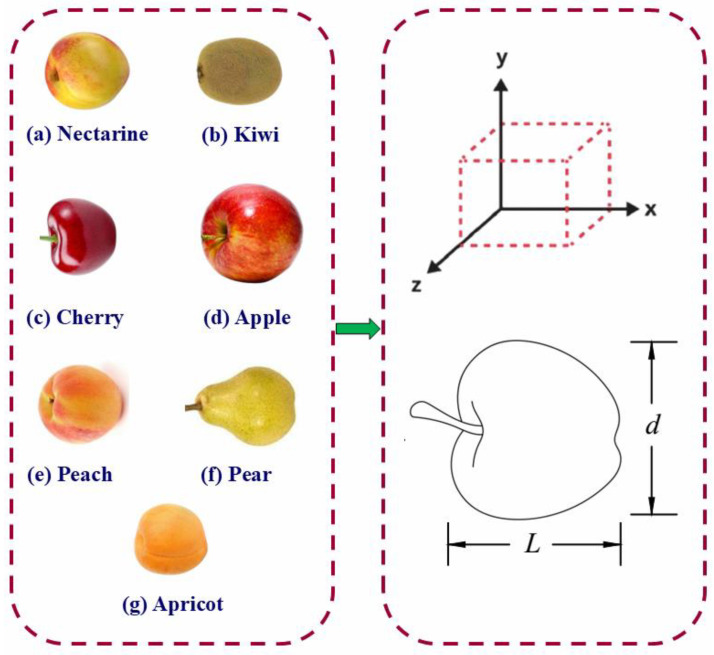
The seven studied fruit types with a representative model illustrating length and diameter.

For all seven fruit types, the physical dimensions were measured using a digital Vernier caliper with an accuracy of 0.05 mm. The measured traits included fruit length (*L*), diameter (*d*), flesh thickness (*Tf*), and pit diameter (*dp*). Figure 1 shows the length and diameter of each fruit. However, for apples, the core diameter was used instead of the pit diameter due to the unique internal structure of the fruit, where the core better represents the central seed cavity than a traditional pit. Additionally, for nectarine, kiwi, and sweet cherry, the fruit diameter (*d*) was calculated as the average of their width and thickness dimensions since these fruits do not closely approximate a spherical shape. Using this averaged diameter provides a more accurate representation of their size compared to using width or thickness alone.

Fruit mass (*m*) was determined using a precision digital balance with a sensitivity of 0.01 g. The volume of each fruit was estimated using the water displacement method as described by Mohsenin [1]. The true density (*ρ_T_*) was then computed as the ratio of fruit mass to its volume [1].

Moisture content (*MC*) was measured for the edible portion (flesh) of each fruit using the standard oven-drying method described by AOAC [22]. To further characterize fruit morphology, the geometric mean diameter (*dg*) was calculated using Equation (1), which considers the length (*L*) and diameter (*d*).

(1) $$d_{g}=\sqrt{L\times d}$$

The sphericity (*S*), which indicates the degree to which a fruit approximates a sphere, was derived using Equation (2) by comparing the geometric mean diameter to the fruit length [1]:

(2) $$S=\frac{d_{g}}{L}$$

Bulk density (*ρb*) was calculated by dividing the total mass of a known volume of fruits by that volume, with the void spaces included, following the protocol established by Mohsenin [1]. All physical measurements were performed on 10 replicates per fruit type to ensure accuracy and represent variability across cultivars.

### 2.3. Mechanical Properties of Fruits

**Figure 2:**
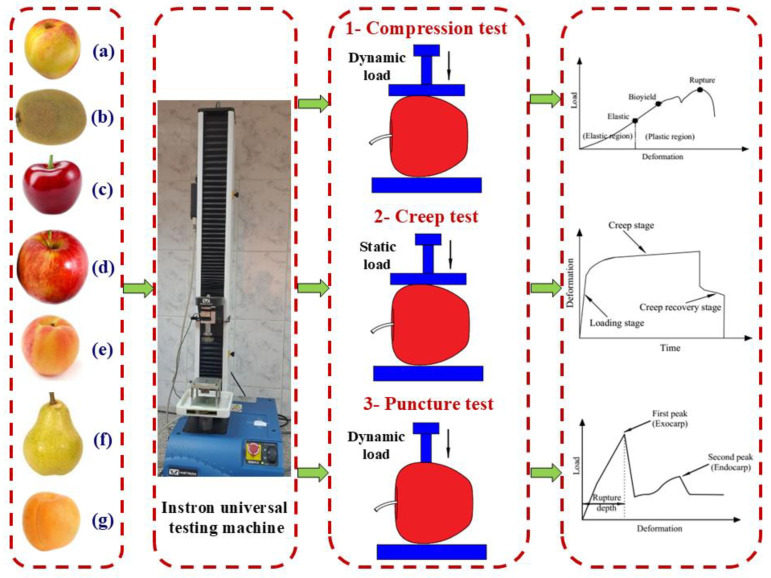
Schematic illustration of the different mechanical tests (compression, creep, and puncture) applied to different fruit samples: (**a**) nectarine, (**b**) kiwi, (**c**) cherry, (**d**) apple, (**e**) peach, (**f**) pear, and (**g**) apricot.

Three different tests were applied to assess the mechanical behavior of fruits under stress conditions: uniaxial compression under a dynamic load, creep under a constant load, and puncture resistance under a dynamic load. Each test was designed to characterize the fruit’s structural response: overall firmness, time-dependent deformation, and resistance to surface rupture. All tests were conducted using a universal testing machine (Instron, 1000 N capacity, Norwood, MA, USA) equipped with interchangeable fixtures and controlled via Bluehill^®^ Universal software (version 4.35; Instron^®^, Norwood, MA, USA). The machine featured a load resolution of 0.01 N and a displacement resolution of 0.001 mm, ensuring high precision in force and deformation measurements. The experiments were performed under displacement control at ambient temperature (23 ± 1 °C) on seven fruit types, each exhibiting distinct anatomical structures and rheological properties. Figure 2 presents a schematic illustration of the mechanical testing procedures and summarizes the general mechanical behavior associated with each test.

#### 2.3.1. Sample Preparation and Environmental Conditioning

Prior to mechanical testing, all fruit samples underwent a standardized conditioning process to stabilize internal moisture distribution and minimize variability due to surface dehydration. The fruits were placed in a climate-controlled chamber maintained at 23 ± 1 °C and 50 ± 5% relative humidity for 24 h. These conditions were chosen to replicate typical ambient environments encountered during storage and transportation in commercial post-harvest handling. Initial height measurements were recorded along the longitudinal axis of each fruit using a digital caliper with an accuracy of 0.01 mm to ensure uniformity in deformation assessment across all replicates.

#### 2.3.2. Compression Test

To evaluate the structural resistance of fruits under compressive loading, a quasi-static uniaxial compression test was conducted on ten randomly selected fruits per type. Each fruit was positioned longitudinally along its natural axis between two parallel flat stainless-steel plates (75 mm diameter). To ensure proper contact and accurate stress distribution, a cylindrical probe was used for smaller fruits, such as cherries. Compression was applied at a constant crosshead speed of 1 mm s^−1^ until visible rupture, significant deformation, or a sudden drop in force was observed. The test was performed under displacement control to record the force–deformation response throughout loading precisely.

The force–deformation curve generated from the test provided key mechanical parameters, including the bioyield force (*Fy*), which indicates the transition from elastic to plastic deformation and is identified by deviation from the initial linear region of the curve, marking the onset of irreversible structural changes. The rupture force (*Fr*) represents the maximum load the fruit can withstand before failure, typically followed by a sharp drop in force. The ratio of rupture to bioyield force (*Rry*), calculated as the ratio of *Fr* to *Fy* (Equation (3)), serves as a normalized measure of structural robustness, where higher values imply greater tolerance to deformation prior to failure.

(3) $$R_{ry}=\frac{F_{r}}{F_{y}}$$

Young’s modulus of elasticity (*E*) was calculated from the slope of the initial linear portion of the force–deformation curve using Equation (4), where *E* is defined as the ratio of stress to strain, with stress (*σ*) calculated as the applied force divided by the cross-sectional area (*F/A*) and strain (*ϵ*) as the deformation relative to the initial height of the fruit. This calculation assumes elastic behavior during the initial loading phase:

(4) E=σϵ=FAΔLLo

where *F* is the applied force, N; *A* is the cross-sectional area of the fruit sample, mm^2^; *ΔL* is the deformation, mm; and *L_o_* is the initial height of the fruit, mm.

The calculation was based on the linear region of the force–deformation curve, assuming elastic behavior during the initial loading phase. Furthermore, the compression work (*W*), representing the total mechanical energy absorbed by the fruit until rupture, was computed as the area under the force–deformation curve up to the rupture point, according to Equation (5). This parameter reflects the fruit’s ability to absorb energy without mechanical failure, an important attribute for soft fruits with high moisture content, such as peaches and apricots:

(5) $$W=\int_{0}^{\delta_{r}}F\left(\delta \right)d\delta$$

where *F(δ)* is the applied force as a function of deformation, N; and *δr* is the deformation at rupture, mm. This parameter is particularly useful for quantifying the fruit’s resistance to mechanical damage and its ability to absorb energy without failing, which is a critical trait for soft and high-moisture fruits like peaches and apricots.

#### 2.3.3. Creep Test

To evaluate viscoelastic behavior, a second set of ten fruits per type was used for creep testing. For each fruit type, a constant force of approximately 20% [23] of the average rupture force observed during compression was applied using the same Instron apparatus. This value was selected based on previous studies [17], which indicate that subcritical loads in the range of 15–25% of rupture force are appropriate for simulating the compressive stresses experienced during storage, stacking, or transportation. This approach allows for capturing realistic mechanical responses without inducing tissue rupture or damage. Fruits were again aligned vertically between the compression plates. Once the target load was reached, the system maintained this force for 300 s, during which the time-dependent displacement was recorded. The deformation curve exhibited two primary phases: instantaneous elastic deformation upon load application and progressive viscous deformation as internal tissues adjusted to the sustained pressure. After the 5 min. loading phase, the compressive force was removed, and the system continued recording the recovery phase for an additional 50 s. This post-load behavior offered insight into residual deformation and the degree of elastic recovery, which vary significantly among fruits due to differences in turgor pressure and cell wall elasticity.

#### 2.3.4. Puncture Test

A third independent set of fruit samples was reserved for puncture resistance testing, which evaluated the peel’s integrity and the outer flesh’s firmness. A 2 mm stainless-steel cylindrical probe was mounted on the Instron machine and driven vertically into the fruit surface at a speed of 1 mm s^−1^. The penetration depth was standardized across all fruit types and determined based on preliminary anatomical assessments conducted on the same sample set, ensuring the probe reached the peel and upper mesocarp without contacting the pit. The initial contact with the fruit surface was detected using a trigger force of 0.1 N. Each fruit was punctured at its equatorial region to minimize anatomical irregularities and maintain consistency between replicates. The force required to penetrate the skin and outer flesh was recorded as the maximum puncture force. The resulting force–displacement curve provided mechanical indicators such as firmness gradient, peel resistance, and tissue rupture strength. Unlike compression and creep tests, which assess whole-fruit behavior, puncture testing is localized and simulates conditions such as skin piercing during harvesting, handling, or mechanical grading. This method is particularly important for delicate fruits such as cherries and apricots, where peel integrity directly affects consumer perception and shelf stability. All instruments, including the texture analyzer and 500 N load cell, were calibrated in accordance with the manufacturer’s guidelines prior to testing.

#### 2.3.5. Instrumentation and Sample Integrity

All mechanical tests were conducted using the same universal testing platform, with only the loading configurations and probe geometries modified for each test type. No sample was reused across multiple tests to maintain the integrity of the results. Fresh, undamaged specimens were selected for each procedure to eliminate cumulative structural effects that could bias the mechanical responses. This approach ensured that each dataset reflected the intrinsic properties of the fruit under the specific testing condition.

### 2.4. Statistical Analysis

Data were analyzed using CoStat (ver. 6.400) with a three-factor randomized complete block design. ANOVA determined the significance of effects and interactions with Duncan’s test for mean comparisons (*p* < 0.05). One- and two-way ANOVA with Tukey’s HSD was used for selected comparisons. Coefficients of variation assessed data consistency, and regression and correlation analyses explored relationships among physical properties [24]. Additionally, Pearson correlation analysis and principal component analysis (PCA) were conducted using IBM SPSS Statistics (v27.0; IBM Corp., Armonk, NY, USA) to evaluate multivariate relationships among the physical and mechanical variables. All multivariate data were standardized prior to analysis [25].

## 3. Results

### 3.1. Physical Properties of Fruits

Table 1 presents the physical characteristics of seven commonly consumed fruit types, including nectarine, kiwi, cherry, apple, peach, pear, and apricot. The analysis included parameters such as length, diameter, flesh thickness, pit diameter, fruit mass, volume, true and bulk density, moisture content, geometric mean diameter, and sphericity. These are essential in assessing their physical and handling behavior during post-harvest operations.

For fruit dimensions, the length and diameter were key measurements in defining the general shape and size of the fruits. Apples and pears displayed the most considerable lengths among the tested fruits, with mean values of approximately 71.13 mm and 81.29 mm, respectively, indicating their elongated morphology, especially in the pear cultivar. Conversely, cherry had the shortest length (around 21.58 mm), consistent with its small, spherical nature. Fruit diameter followed a similar trend, with apples reaching a maximum average of 76.96 mm. Notably, nectarine and kiwi demonstrated very close values of length and diameter (e.g., nectarine: ~63.5 mm length and ~64.1 mm diameter), which support their near-spherical form. The sphericity values, calculated based on the geometric mean diameter, further confirmed this, with nectarine exhibiting a high sphericity (~1.00), indicating minimal deviation between the axial dimensions. At the same time, kiwi showed a relatively lower value (~0.89), suggesting more elongation. Flesh thickness (*Tf*) is particularly significant for mechanical property studies, especially in determining fruit firmness and suitability for non-destructive testing. Among the fruits evaluated, nectarines had the highest average flesh thickness (~18.96 mm), followed by pears and apples (~12–12.7 mm). In contrast, cherries showed the lowest flesh thickness (~7.12 mm), reflecting their smaller size and higher pit-to-flesh ratio. These values are critical when considering consumer acceptability and potential peeling or slicing operations [26,27].

Regarding pit diameter (*dp*), apples and peaches had values exceeding 22 mm, while cherries and kiwi had substantially smaller pits (below 15 mm). Notably, apples’ core diameter (22.97 mm) aligned closely with previous studies on ‘Golden Delicious’ and ‘Red Chief’ cultivars [28]. This parameter influences the edible portion and is essential for evaluating bioyield and mechanical damage during coring. Apples exhibited the highest average fruit mass (~160.70 g), followed closely by pears and nectarines. Cherries, as expected, had the lowest mass (~7.84 g). These weight differences significantly affect packaging and transportation design. The volume measurements showed a proportional relationship with fruit mass, indicating consistent density across the fruit tissues. Notably, pear and apple fruits had the highest volumes (>165 cm^3^), whereas cherries were again the smallest (~6.55 cm^3^). True density values (*ρ_T_*), calculated as mass-to-volume ratios, revealed that cherries had the highest density (~1.21 g cm^−3^), indicating a more compact structure, while apples had the lowest (~0.95 g cm^−3^). These findings are consistent with recent observations in fruit characterization studies [29,30].

Bulk density (*ρb*), which considers the voids between fruits in bulk storage or packaging, was generally lower across all fruit types, with a uniform value of ~0.60 g cm^−3^ in apple, pear, peach, and apricot. Cherries again exhibited the highest bulk density (~0.55 g cm^−3^), likely due to their small size allowing for denser packing. These values are essential for designing storage bins and optimizing load capacities in transportation [31]. Moisture content (*MC*) across all fruit types remained consistently high, ranging from approximately 83% to nearly 87%, reflecting the fruits’ fresh state at full ripeness. Peaches showed the highest average moisture content (~86.96%), corresponding to their soft texture and juicy characteristics. Conversely, nectarines and kiwis displayed slightly lower moisture levels (~83.3–83.8%), still within the acceptable range for fresh consumption but indicating more compact flesh. These findings align with previous assessments of post-harvest moisture variability in stone and pome fruits [32]. Geometric mean diameter (*dg*), calculated using the formula involving length and diameter, was highest in apples (~73.98 mm), followed by pears and nectarines. Cherries had the smallest geometric mean (~22.79 mm), supporting the morphological assessments. The geometric mean is particularly valuable for predicting rolling behavior on conveyor systems and aids in size grading processes [33]. Sphericity (*S*) provides insight into the degree of roundness of the fruits. Most fruits, including apples, peaches, and apricots, had high sphericity values (>1.00), indicating near-spherical shapes. Kiwi had the lowest sphericity (~0.89), suggesting an elongated structure. This characteristic is important in determining fruit orientation during automated handling or packaging. Recent studies have reported similar sphericity trends involving non-destructive imaging and shape recognition in fruits [34]. Several of these physical properties influence the mechanical response and post-harvest behavior of the fruits. For instance, thicker pulp (*Tf*) may allow greater energy absorption under compression, reducing rupture risk, while higher fruit mass and volume can affect stacking pressure tolerance during storage. Variations in density also reflect tissue compactness, which plays a role in firmness and deformation capacity.

Overall, the physical characteristics observed for these fruits provide critical inputs for designing handling, storage, and processing equipment. The moderate to high variability in some traits, such as pit diameter and flesh thickness, suggests that cultivar-specific adaptations may be necessary. Statistical analysis confirmed significant differences (*p* < 0.05) between the fruit types across most measured traits, reinforcing the importance of tailored post-harvest strategies for each fruit species.

### 3.2. Mechanical Properties of Fruits

#### 3.2.1. Fruit Compression Test

**Figure 3:**
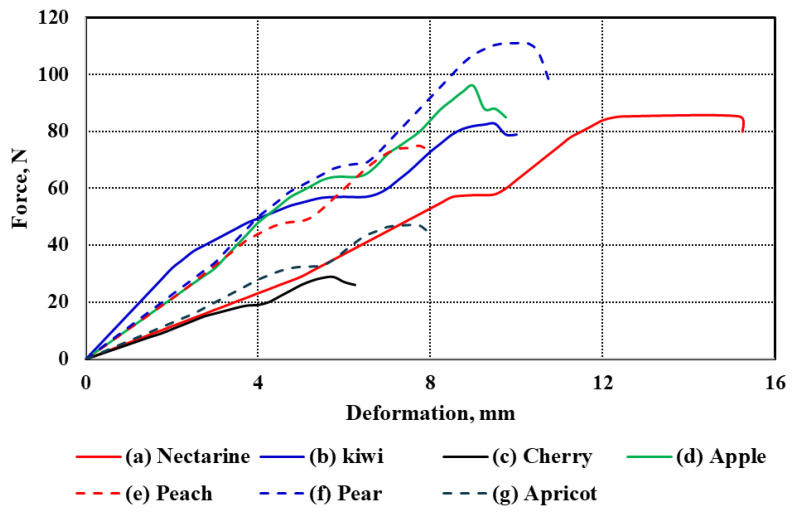
Force–deformation curves of the seven fruit types under compression test.

The force–deformation curves of the seven studied fruit types under uniaxial compression are illustrated in Figure 3a–g. These curves represent the mechanical response of each fruit under compressive loading, revealing distinct patterns that reflect differences in tissue structure, firmness, and anatomical features. All curves follow a typical biological material response, starting with an initial elastic region, then a nonlinear plastic deformation phase, finally leading to rupture and fracture. While the general trend is consistent across the fruits, notable differences exist in the extent and shape of the deformation profiles.

Among all samples, cherry exhibited the lowest deformation values across all stages, which aligns with its small size and relatively thin flesh (*Tf* = 7.12 mm). In contrast, nectarine showed the highest deformation range, extending beyond 15 mm, consistent with its relatively large diameter (*d* = 64.11 mm) and moderate flesh thickness (*Tf* = 18.96 mm). Similarly, pear and apple, which have substantial sizes (*L* = 81.29 mm and 71.13 mm, respectively), demonstrated extended deformation curves, suggesting more gradual energy absorption before failure.

The kiwi and apricot curves exhibit intermediate behavior. Although kiwi has a smaller diameter (*d* = 50.24 mm) than nectarine and apple, its relatively thick flesh (*Tf* = 12.02 mm) appears to contribute to its moderate resistance to deformation. Despite having a smaller size (*L* = 36.17 mm, *d* = 36.37 mm), apricot still demonstrates appreciable deformation capacity, likely influenced by its moderately thick pericarp (*Tf* = 9.38 mm). On the other hand, peach, with a diameter comparable to nectarine (*d* = 65.21 mm), showed a shorter deformation span, which may be attributed to its lower flesh thickness (*Tf* = 9.25 mm) and potential structural differences in cell arrangement. Apple and pear exhibited the most extended rupture regions, reflecting their firm internal structure and high resistance to mechanical damage. Their deformation curves also show a more gradual decline after peak force, indicating a more ductile-like failure compared to the sharper drop observed in fruits like cherries and apricots. These differences are likely influenced by fruit size, geometry, and tissue composition, such as cell wall thickness, intercellular adhesion, and moisture content.

Overall, the observed differences in deformation behavior among the fruits can be attributed to the combined effects of morphological parameters—length (*L*), diameter (*d*), and flesh thickness (*Tf*)—as summarized in Table 1. Larger and thicker-fleshed fruits generally showed higher deformation capacities before rupture, whereas smaller and thinner-fleshed fruits exhibited earlier failure. These mechanical characteristics are critical for post-harvest handling, packaging, and processing decisions, where fruit resistance to mechanical stress directly impacts shelf life and market quality.

The mechanical parameters evaluated for the studied fruits, as presented in Table 2, include bioyield force (*Fy*), rupture force (*Fr*), the ratio of rupture to bioyield force (*Rry*), modulus of elasticity (*E*), and the work required to rupture (*W*). These properties reflect each fruit type’s texture, structural integrity, and resistance to mechanical damage. Bioyield force and rupture force showed noticeable variations among the fruits. Pear and apple recorded the highest values in both parameters, indicating a firmer and more resistant structure. In contrast, cherry and apricot had the lowest forces, highlighting their delicate texture. For example, the rupture force of the pear exceeded 110 N, while cherry registered values below 30 N, showing a clear contrast in mechanical strength. Nectarine, kiwi, and peach showed intermediate behavior, with rupture forces ranging from the mid-70s to mid-80s N, suggesting they possess moderate firmness compared to the two extremes. The ratio of rupture to bioyield force values for most fruits were relatively close, generally ranging from 1.45 to 1.62. Pear had the highest strain, indicating a greater ability to deform before rupture, while fruits such as kiwi and apricot showed slightly lower strain values. These results suggest that most fruits exhibited a certain level of elasticity before structural failure, although the extent of this elasticity varied slightly among them. The modulus of elasticity, reflecting the stiffness of fruit tissues, varied noticeably among the tested samples. Kiwi demonstrated the highest stiffness with an elasticity modulus of 0.72 MPa, followed by pear, apricot, and apple, all of which showed moderate values around 0.36–0.37 MPa.

In contrast, nectarine and peach exhibited lower stiffness values, approximately 0.28–0.29 MPa, while cherry remained on the lower end with a modulus of 0.40 MPa. These differences highlight the variation in structural firmness among fruits, which is influenced by their internal cellular composition and water content. The work required to rupture the fruit, representing the energy absorbed during deformation and fracture, followed trends consistent with the force values. Pear required the highest energy to rupture, with a value of around 0.72 kJ, while cherry absorbed the least energy, approximately 0.31 kJ. Apple and peach also required relatively high energy inputs, ranging between 0.58 and 0.65 kJ, suggesting that firmer fruits resist rupture more effectively. In contrast, apricots and nectarines required moderate energy inputs, reflecting their intermediate firmness.

Statistical analysis confirmed that differences among fruit types were significant across all mechanical parameters (*Fy*, *Fr*, *Rry*, *E*, and *W*). Pear consistently exhibited the highest values, significantly differing from most other fruits—particularly cherry and apricot—regarding bioyield force, rupture force, rupture-to-bioyield ratio, and energy to rupture. Apple also showed high mechanical strength and was statistically superior to softer fruits like cherry and apricot. However, it was not always significantly different from intermediate-texture fruits such as nectarine and peach. Nectarine and kiwi often appeared within the same statistical group, suggesting their mechanical properties are comparable, especially in bioyield force and rupture characteristics. In contrast, cherry and apricot consistently formed the lowest statistical groups across most parameters, confirming their relatively soft texture and vulnerability to mechanical damage.

These findings underline the broad mechanical diversity among fruit types. Firmer fruits like pear and apple, with higher resistance to rupture and energy absorption before failure, are better suited for handling and transport. Meanwhile, softer fruits such as cherries and apricots require more delicate post-harvest treatment to reduce mechanical injury and preserve quality.

#### 3.2.2. Fruit Creep Test

**Figure 4:**
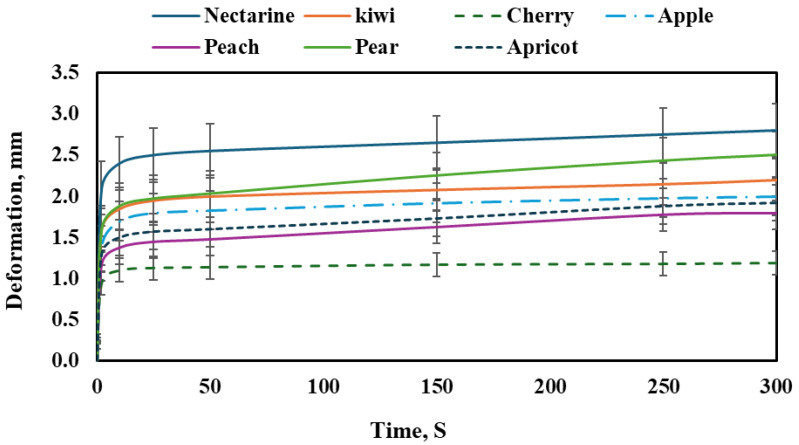
Creep behavior of selected fruit under a constant load over time.

Figure 4 shows the creep behavior of different fruit types under a constant load over time. All fruits demonstrated a time-dependent increase in deformation, characteristic of viscoelastic materials. The general trend reveals an initial rapid deformation phase followed by a slower, more stable increase known as primary and secondary creep. However, the extent of deformation varied significantly between the fruits, indicating differences in tissue firmness, internal structure, and moisture content.

Nectarine exhibited the highest deformation among all tested fruits, reaching approximately 2.85 mm by the end of the test period. The curve shows a rapid initial increase, followed by a gradual but steady rise, indicating relatively soft tissue with sustained susceptibility to creep over time. This behavior suggests a low internal resistance to long-term deformation.

The kiwi followed a similar pattern but with slightly less overall deformation. Its final deformation was about 2.3 mm, and the curve remained stable after the initial sharp increase, reflecting a tissue that, while soft, begins to resist additional deformation beyond the primary phase. This suggests moderate firmness compared to nectarine.

The cherry demonstrated the lowest deformation of all fruits, reaching a maximum of only 1.25 mm. The creep curve rose sharply in the initial seconds but then flattened significantly, showing minimal deformation throughout the rest of the test. This behavior indicates a firmer tissue structure and higher resistance to creep, possibly due to dense skin and a strong internal matrix. The apple showed moderate creep behavior, with the deformation reaching around 2.1 mm. The curve exhibited a typical viscoelastic pattern, with a fast initial increase followed by a much slower rate. The apple’s relatively low creep compared to softer fruits suggests a balance between elasticity and tissue rigidity. The peach exhibited a deformation curve that reached approximately 1.9 mm at the end of the testing period. Its behavior was similar to the apple’s, although the final deformation was slightly lower. The initial phase showed a steep increase in strain, followed by stabilization, indicating soft but structured tissue.

The pear demonstrated one of the higher deformation values, with a final strain of about 2.6 mm. Its curve indicates sustained creep beyond the initial phase, suggesting a softer tissue structure than initially expected, possibly due to high moisture content or cellular degradation that allows greater deformation under stress. The apricot showed a final deformation close to 1.8 mm. The pattern followed the typical viscoelastic trend with a quick rise in the beginning, and then a slower, more stable increase. Its performance is in the moderate range, showing more resistance than nectarine and pear but less than cherry and apple.

Among all the fruits, certain varieties showed comparable behavior. For instance, nectarine and pear exhibited similarly high deformation values, indicating soft and less resistant tissues. On the other hand, cherry and apricot showed relatively low creep, suggesting firmer textures. Apple and peach displayed moderate creep behavior and can be considered mechanically similar in resistance to time-dependent deformation. These observed differences in creep deformation among the fruit types were statistically significant (*p* < 0.05), as confirmed by ANOVA.

These creep responses can be further interpreted considering the mechanical properties measured during compression testing (Table 2). Since the applied load in the creep test was standardized at approximately 20% of each fruit’s average rupture force, the resulting deformations reflect the strength and how each tissue tolerates prolonged subcritical stress. The elastic modulus, rupture force, and required work to rupture are particularly relevant here, as they describe the tissue’s immediate resistance, rigidity, and energy absorption capacity.

Nectarine, which had a relatively low modulus of elasticity (0.28 MPa) and moderate rupture force (85.4 N), showed the highest creep deformation. This indicates limited structural stiffness and a pronounced susceptibility to progressive strain under constant load. A somewhat similar pattern was observed in pear, which, despite having the highest rupture force (111.1 N), had a moderate modulus of elasticity (0.36 MPa) but still underwent substantial creep. This apparent contradiction may be attributed to internal factors such as elevated turgor pressure or loosely packed tissue microstructure, which facilitate deformation over time. In contrast, cherry exhibited minimal creep deformation despite its low rupture (28.8 N) and bioyield (18.8 N) forces, along with a modest elastic modulus of 0.40 MPa. This suggests that factors beyond fundamental mechanical strength—such as cell compactness, cuticle thickness, or anatomical arrangement—play a significant role in resisting time-dependent deformation. Fruits such as apple and apricot, with similar elastic modulus values (0.36 and 0.37 MPa, respectively) and comparable rupture strengths, displayed creep behavior indicative of balanced firmness: not highly resistant, but also not prone to excessive deformation. Peach, with a slightly lower modulus (0.29 MPa), followed a similar trend, while kiwi, which had the highest modulus of elasticity (0.72 MPa), showed relatively restrained creep deformation, consistent with its greater tissue stiffness and structural integrity. Integrating creep data with compression-derived mechanical properties reveals a more nuanced understanding of fruit texture. While rupture force and elastic modulus offer insight into how fruits resist instantaneous loading, creep analysis uncovers how these tissues behave under prolonged mechanical stress. Such dual perspectives are essential for characterizing the viscoelastic nature of biological materials and have practical implications for post-harvest handling, packaging, and shelf-life prediction.

#### 3.2.3. Fruit Puncture Test

**Figure 5:**
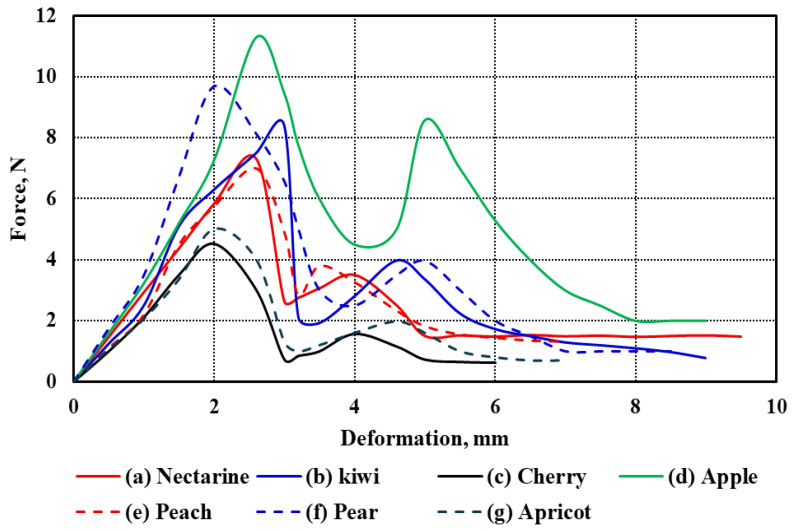
Puncture behavior of fruit types.

Figure 5 illustrates the fruit puncture behavior for the seven studied fruit types, showing both the puncture force and the corresponding deformation for the exocarp (outer skin) and endocarp (inner flesh around the seed or pit). The results highlight substantial differences in mechanical resistance and structural integrity across fruit types.

Apple exhibited the highest puncture force values among all tested fruits. The exocarp required a force of 11.3 N to puncture, with a deformation of 2.8 mm, indicating a tough and elastic outer skin. For the endocarp, the puncture force was also significantly high at 8.6 N, with a deformation of 4.8 mm. This reflects a consistent internal structure capable of resisting mechanical stress. The apple’s dense, turgid parenchyma and relatively thick cuticle contribute to its high resistance. These characteristics make apples more resilient during harvesting, transportation, and storage, reducing the risk of bruising. Pear showed a similar trend to apple in terms of mechanical strength. The exocarp puncture force was 9.7 N with 2.7 mm deformation, indicating considerable stiffness. The endocarp required 3.9 N and exhibited a 4.9 mm deformation. While the external layer shows strength close to that of the apple, the lower internal resistance and slightly greater deformation suggest a softer flesh. Pears, like apples, are classified as pome fruits, which explains the similarity in structural properties.

However, the increased softness of the endocarp may affect their susceptibility to internal bruising despite their tough skin. Kiwi displayed relatively high values of puncture resistance as well. The exocarp required a force of 8.4 N and deformed by 2.9 mm, while the endocarp needed 3.36 N with a deformation of 5.1 mm, which was the highest deformation among all fruits in the endocarp. This highlights a significant discrepancy between the firm outer skin and the soft, highly deformable interior. Kiwi’s endocarp comprises loosely packed, water-rich tissue, contributing to its high deformability. This duality indicates that while the skin offers good initial protection, the inner tissue is susceptible to pressure, requiring careful handling. Nectarine had a moderately resistant exocarp, requiring 7.1 N with a deformation of 2.6 mm. The endocarp, by contrast, needed only 2.9 N to puncture, with a deformation of 4.6 mm. These values suggest moderately firm skin and a relatively soft inner layer.

Nectarines, being stone fruits, have a thin cuticle and a juicy mesocarp, which can be easily damaged during handling. The moderate puncture resistance in both layers places nectarines in the middle range regarding mechanical vulnerability. Peach, closely related to nectarine, showed very similar puncture behavior. The exocarp puncture force was 7.0 N with 2.4 mm deformation, and the endocarp required 2.31 N with 4.5 mm deformation. These values suggest that peaches are only slightly softer than nectarines. Given their structural similarities—both are Prunus species with large pits and similar textures—the mechanical response is expectedly close. However, even slight differences in firmness can impact storage life and transportability.

Apricot demonstrated lower puncture force values compared to other stone fruits. The exocarp puncture force was 5.9 N with a deformation of 2.2 mm, while the endocarp required 1.9 N with 4.6 mm deformation. Although the deformation values are not significantly different from those of nectarine or peach, the lower force values indicate weaker tissue resistance. This makes apricots more prone to surface and internal damage, necessitating more delicate post-harvest handling conditions. Cherry had the lowest puncture resistance among all fruits. The exocarp required only 4.5 N with the least deformation at 1.9 mm. The endocarp showed the lowest force value (1.5 N) and a deformation of 4.1 mm. These results reflect the delicate structure of cherries, which have thin skin and very soft pulp. The small size and fragile tissue make cherries extremely sensitive to mechanical damage, often requiring specialized packaging and minimal handling.

Upon comparing the data, fruits such as apples and pears stand out for their high puncture resistance in both exocarp and endocarp, making them suitable for more extended storage and transport. In contrast, cherry and apricot are at the opposite end of the spectrum, with very low resistance, requiring careful handling. The stone fruits—nectarine, peach, and apricots—show similar deformation behavior but differ slightly in force values, with apricot being the weakest. Meanwhile, kiwi presents a unique case with a firm exterior but extremely soft interior, indicating a high risk of internal damage if mishandled. These observations are critical for post-harvest applications, as they inform decisions related to harvesting tools, packaging design, and transport methods tailored to each fruit’s mechanical profile.

### 3.3. PCA and Correlation Analysis of Physical–Mechanical Relationships

**Figure 6:**
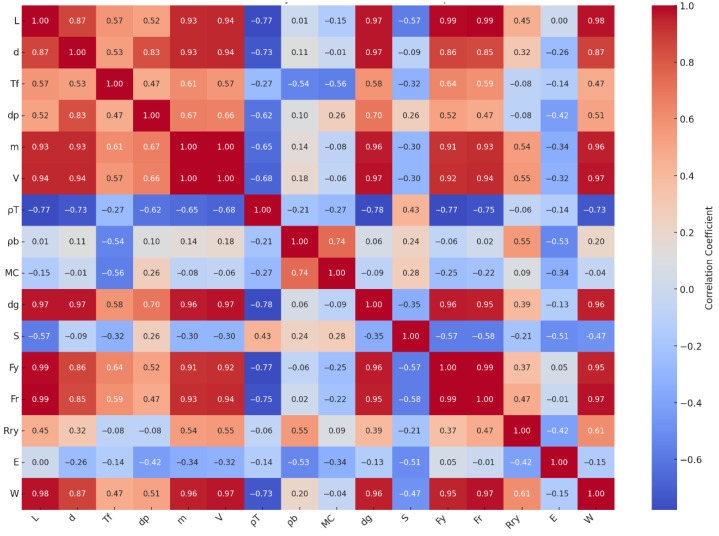
Correlation matrix illustrating the relationships between physical and mechanical properties of the studied fruit types.

The principal component analysis (PCA) revealed clear patterns of association between the physical and mechanical properties of the examined fruits. The first two components explained a substantial portion of the total variance, allowing a meaningful interpretation of the relationships among traits. As shown in Figure 6, size- and mass-related variables such as fruit length, diameter, mass, volume, and geometric mean diameter were closely aligned with mechanical strength indicators including rupture force, bioyield force, and the work required to rupture. This suggests that larger fruits with greater mass tend to exhibit higher mechanical resistance, likely due to their thicker peel and denser structural tissues which provide greater support under compression.

In contrast, internal characteristics such as moisture content and bulk density showed a distinct influence, particularly on properties related to elasticity and time-dependent deformation. Fruits with higher moisture content tend to exhibit lower rupture strength but increased flexibility, as water softens cell walls and reduces structural cohesion. This inverse relationship aligns with the contribution of moisture to the second principal component, where modulus of elasticity was also influential. Thus, while size enhances strength, water content modulates deformability.

Elastic modulus, unlike other force-related properties, showed limited association with size but was more strongly influenced by moisture, density, and tissue structure. This reflects the fact that elasticity arises from the internal cellular arrangement and hydration status rather than external dimensions alone. Similarly, creep behavior, although not a primary factor in the PCA, may be indirectly related to moisture and bulk density. Fruits with higher water content and less compact structure are more likely to exhibit time-dependent deformation under load due to weaker internal bonding.

**Figure 7:**
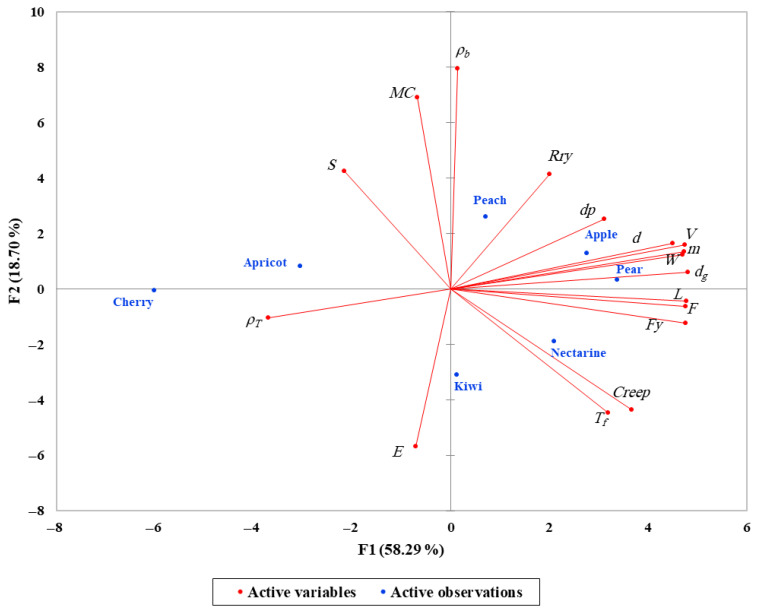
Principal component analysis (PCA) biplot illustrating the relationship between physical and mechanical properties of different fruits.

These results highlight that the mechanical response of fruit tissue is not governed by a single attribute but by a combination of physical form, compositional features, and internal architecture. While mass and volume increase the force required to rupture the fruit, parameters such as moisture content and density shape the elastic and viscoelastic behavior. The spatial distribution of fruit types in Figure 7 reinforces these interactions; for instance, apple, pear, and peach occupy regions corresponding to high mass and strength, whereas cherry and apricot are associated with lower structural resilience. Kiwi and nectarine exhibit intermediate behavior, reflecting moderate mechanical resistance and moisture-related flexibility.

Moreover, the relative positions of the fruit samples in the PCA plot offer insight into their overall similarity or divergence. Fruits that are located near each other in the PCA space—such as kiwi and nectarine—share comparable combinations of physical and mechanical traits. In contrast, distant placements—for example, between apple and apricot—indicate substantial differences in their structural properties. Therefore, PCA not only clarifies trait interactions but also serves as an effective tool for visually identifying groups of fruits with similar quality characteristics, which can inform classification, grading, and post-harvest handling decisions.

Additionally, Figure 6 presents the correlation matrix between the physical and mechanical properties of the studied fruit types, offering a clear visualization of key interactions. To further explore these relationships, a Pearson correlation analysis was conducted across all fruits. The results revealed strong positive correlations between size-related physical attributes—such as fruit length (*L*), diameter (*d*), geometric mean diameter (*d_g_*), and fruit mass (*m*)—and mechanical strength indicators including bioyield force (*F_y_*), rupture force (*Fr*), and the work required to rupture (*W*). For example, fruit mass showed high correlation coefficients with *F_y_* (r = 0.91), *F_r_* (r = 0.93), and *W* (r = 0.96), suggesting that heavier fruits possess a more robust structural resistance under compressive loading. Similarly, geometric attributes like *L* and *dg* were also closely linked to these force parameters (r > 0.85), supporting the notion that physical size substantially contributes to mechanical resilience. Moreover, moisture content (*MC*) was found to negatively correlate with modulus of elasticity (*E*) (r = –0.34), implying that fruits with higher water content tend to exhibit lower stiffness and are more prone to deformation, consistent with previously reported viscoelastic behavior. Bulk density (*ρb*) also showed a moderate negative correlation with *E* (r = –0.53), further indicating that lower-density tissues may deform more under sustained loads. Flesh thickness (*Tf*), while moderately correlated with strength traits like *Fy* (r = 0.64) and *Fr* (r = 0.59), also plays a role in energy absorption capacity, as reflected in its relationship with *W* (r = 0.47).

These findings quantitatively reinforce the mechanical interpretations derived from PCA and support the hypothesis that fruit mechanical behavior is governed not only by macroscopic dimensions but also by internal water content and structural compactness. The integration of univariate correlation analysis with multivariate PCA thus provides a more comprehensive understanding of the physical–mechanical interplay affecting post-harvest fruit quality.

## 4. Discussion

In this study, the comprehensive evaluation of selected fruits’ physical and mechanical properties was undertaken to understand their integrated role in post-harvest quality and handling optimization. The overarching goal was to establish intrinsic physical traits such as size, mass, flesh thickness, and moisture content in conjunction with mechanical characteristics like rupture force, elasticity, and creep behavior, which affect fruit resistance to damage, storability, and processing suitability. These traits directly influence decisions regarding harvesting methods, sorting protocols, packaging design, transportation resilience, and consumer satisfaction. The physical properties analyzed—such as fruit dimensions, mass, volume, true and bulk density, and moisture content—represent fundamental fruit structure and internal composition indicators. Larger fruits such as apples and pears exhibited greater mass and volume and thicker flesh layers, contributing to their higher geometric mean diameters and sphericity. These attributes play a critical role in mechanical performance. For instance, thicker mesocarp tissue, as seen in nectarines (~18.96 mm), provides more cushioning during compression, leading to higher deformation capacity without immediate rupture. Moisture content, which ranged from ~83% in nectarines to ~87% in peaches, significantly influenced the viscoelastic behavior of the fruits. High moisture levels are associated with lower stiffness and increased susceptibility to creep and rupture, a pattern observed in peaches and apricots. This aligns with findings from Zhang et al. [35], who demonstrated that higher water content reduces cell wall rigidity and increases turgor-driven deformation, especially under sustained load or pressure.

In mechanical resistance, fruits like apples and pears—characterized by high mass, moderate sphericity, and lower moisture content—exhibited the highest bioyield and rupture forces. This resilience makes them ideal for mechanized harvesting, where fruits are subjected to shaking or impact forces, and for bulk transportation, where stacking pressure can be significant [36,37]. By contrast, the lower rupture force and modulus of elasticity in cherries and apricots reflect their vulnerability and confirm their need for manual harvesting, customized small-volume packaging, and minimized handling to avoid mechanical injury. The modulus of elasticity, which indicates fruit stiffness, was highest in kiwi and lowest in nectarine and peach. This parameter is critical for automated sorting systems that rely on firmness detection. Fruits with low elasticity often suffer from misclassification or excessive pressure during mechanical sorting, leading to bruising and quality deterioration [38,39]. Creep tests, simulating long-term storage conditions, further highlighted the importance of tissue structure in resisting time-dependent deformation. Nectarine and pear showed the most significant deformation under sustained subcritical load, indicating susceptibility to shape distortion during storage [40]. This compromises visual appeal and increases the likelihood of fungal infections through compromised tissues. Cherry, however, showed minimal creep deformation, suggesting a compact cellular structure and higher recovery ability for maintaining shape integrity during extended shelf display [41].

The puncture test, crucial for understanding peel integrity, revealed that fruits with thicker or denser exocarps (apple, pear) offer higher protection against mechanical piercing. This makes them better suited for long-distance trade and automated grading systems [42]. Conversely, fruits like cherries and apricots with lower puncture resistance are more susceptible to cuticle damage, leading to accelerated spoilage. Moreover, mechanical behavior correlates with processing suitability [6]. For instance, softer fruits with low rupture energy, like peach and apricot, are favorable for juicing and pulping, where lower mechanical input yields better extractability. In contrast, firmer fruits with higher energy absorption (e.g., pear, apple) are more suitable for drying or slicing, maintaining shape and texture post-processing [14,43].

From a quality assurance perspective, integrating physical and mechanical data enhances the predictive modeling of fruit durability and shelf life. Ghonimy et al. [3] showed that rupture force and elastic modulus can serve as key indicators for estimating fruit toughness and damage thresholds during packaging and transport simulations. Furthermore, non-destructive technologies like acoustic or tactile sensors benefit from such foundational data to improve accuracy in firmness grading and ripeness detection [44]. These interactions between fruit size, moisture, and mechanical strength are further corroborated by the multivariate structure revealed by the PCA results, which underscores the joint influence of both morphological and compositional attributes on the mechanical response of fruit tissues. While this study offers a detailed characterization of the physical and mechanical properties of seven fruit types, it is important to acknowledge certain limitations. The analysis was confined to macro-scale mechanical testing under controlled conditions. Aspects such as microstructural anatomy, biochemical composition, and sensory quality—including taste and texture perception—were beyond the scope of this investigation. Additionally, real-world dynamic stresses such as vibration and impact during handling and transport were not assessed. These factors are essential for a more holistic understanding of post-harvest fruit behavior and are recommended for future research on maintaining fruit quality.

Furthermore, this study focused exclusively on fruits harvested at commercial maturity to ensure standardization and practical relevance. However, it is recognized that fruit characteristics may vary with seasonal conditions and at different physiological ripening stages.

## 5. Conclusions

The integrated analysis of physical and mechanical properties presented in this study reinforces the critical importance of these parameters in determining overall fruit quality and guiding post-harvest operations. Fruit quality is not defined solely by visual or nutritional traits. However, it is strongly influenced by structural and mechanical attributes that dictate how fruits respond to external forces during handling, storage, and processing. Traits such as firmness, elasticity, peel resistance, and viscoelastic behavior directly affect critical quality indicators like texture retention, shape preservation, shelf life, and consumer appeal. These characteristics are essential for designing efficient and safe post-harvest handling systems. Fruits with higher mechanical strength are better suited to automated harvesting, mechanical sorting, bulk packaging, and extended cold storage. Conversely, fruits with lower resistance require protective handling, custom cushioning, and often manual intervention to avoid quality degradation. The data also inform decisions in downstream processes such as slicing, drying, juicing, and fresh-cut preparation, where mechanical integrity influences yield, energy use, and final product uniformity. By bridging physical descriptors with mechanical performance, the study offers a practical framework for optimizing post-harvest strategies based on fruit-specific behavior. This approach supports quality assurance at all value chain stages and reduces mechanical injuries, post-harvest losses, and consumer dissatisfaction. Future research may expand on this work by incorporating microscopic tissue imaging and sensory evaluation, which would provide a more comprehensive view of structural and consumer-relevant attributes. Also, future studies should investigate how such factors influence mechanical performance and post-harvest quality. It should be noted that all fruits used in this study were grown under Egyptian climatic conditions; therefore, results may vary under different agro-ecological settings.

## Figures and Tables

**Table 1:** Physical characteristics of seven fruit types (mean ± SD), *n* = 10.

Property	Nectarine	Kiwi	Cherry	Apple	Peach	Pear	Apricot
*L*, mm	63.51 ± 1.66	63.66 ± 2.87	21.58 ± 1.96	71.13 ± 3.21	60.82 ± 3.10	81.29 ± 6.00	36.17 ± 1.05
*d*, mm	64.11 * ± 1.04	50.24 * ± 2.12	24.10 ± 1.67	76.96 ± 3.13	65.21 ± 2.97	59.50 ± 2.73	36.37 ± 1.54
*T_f_*, mm	18.96 ± 1.33	12.02 ± 0.55	7.12 ± 0.59	11.43 ± 0.47	9.25 ± 0.53	12.70 ± 1.43	9.38 ± 0.21
*dp*, mm	22.74 ± 1.59	14.02 ± 1.12	6.65 ± 0.45	22.97 ** ± 1.51	23.48 ± 0.93	12.94 ± 1.32	16.35 ± 0.85
*m*, g	151.12 ± 6.34	80.51 ± 3.78	7.84 ± 0.70	160.70 ± 14.34	136.57 ± 8.40	173.30 ± 12.22	40.99 ± 4.88
*V*, cm^3^	142.40 ± 4.90	82.40 ± 4.20	6.55 ± 0.69	169.10 ± 14.59	136.50 ± 7.85	175.20 ± 11.99	40.53 ± 4.50
*ρ_T_*, g cm^−3^	1.06 ± 0.01	0.98 ± 0.06	1.21 ± 0.18	0.95 ± 0.02	1.00 ± 0.03	0.99 ± 0.02	1.01 ± 0.03
*ρ_b_*, g cm^−3^	0.48 ± 0.03	0.47 ± 0.04	0.55 ± 0.03	0.60 ± 0.04	0.60 ± 0.04	0.60 ± 0.04	0.60 ± 0.04
*MC*, %	83.28 ± 1.29	83.80 ± 0.64	84.14 ± 1.63	84.64 ± 1.03	86.96 ± 0.87	84.64 ± 0.92	86.47 ± 1.40
*d_g_*, mm	63.80 ± 1.29	56.55 ± 2.44	22.79 ± 1.58	73.98 ± 3.04	62.96 ± 2.76	69.52 ± 3.83	36.26 ± 1.16
*S*	1.00 ± 0.01	0.89 ± 0.01	1.06 ± 0.04	1.04 ± 0.01	1.04 ± 0.02	0.86 ± 0.02	1.00 ± 0.02

*L* = Fruit length, *d* = fruit diameter, *T_f_* = flesh thickness, *dp =* pit diameter, *m* = fruit mass, *V* = fruit volume, *ρ_T_* = true density, *ρ_b_* = bulk density, *MC* = moisture content, *d_g_* = geometric mean diameter, *S* = degree of sphericity. * Average diameter, ** core diameter for apple.

**Table 2:** Mechanical properties of selected fruits under compression testing.

Fruit	*Fy*, N	*Fr*, N	*Rry*	*E*, MPa	*W*, kJ
Nectarine	57.7 ^bc^	85.4 ^c^	1.48 ^b^	0.28 ^bc^	0.55 ^bc^
Kiwi	57.1 ^bc^	82.8 ^c^	1.45 ^b^	0.72 ^a^	0.52 ^c^
Cherry	18.8 ^d^	28.8 ^e^	1.52 ^ab^	0.40 ^b^	0.31 ^d^
Apple	64.1 ^ab^	98.1 ^b^	1.53 ^ab^	0.36 ^b^	0.65 ^ab^
Peach	48.2 ^c^	74.3 ^d^	1.54 ^ab^	0.29 ^bc^	0.58 ^bc^
Pear	68.6 ^a^	111.1 ^a^	1.62 ^a^	0.36 ^b^	0.72 ^a^
Apricot	32.6 ^cd^	47.2 ^d^	1.45 ^b^	0.37 ^b^	0.39 ^d^

*Fy* = bioyield force, *Fr* = rupture force, *Rry* = ratio of rupture to bioyield force, *E* = modulus of elasticity, and *W* = work required to rupture. Different letters indicate significant differences (*p* < 0.05) within each column.

## Data Availability

The original contributions presented in this study are included in the article. Further inquiries can be directed to the corresponding author.
